# RUNX2 correlates with subtype-specific breast cancer in a human tissue microarray, and ectopic expression of *Runx2* perturbs differentiation in the mouse mammary gland

**DOI:** 10.1242/dmm.015040

**Published:** 2014-03-13

**Authors:** Laura McDonald, Nicola Ferrari, Anne Terry, Margaret Bell, Zahra M. Mohammed, Clare Orange, Alma Jenkins, William J. Muller, Barry A. Gusterson, James C. Neil, Joanne Edwards, Joanna S. Morris, Ewan R. Cameron, Karen Blyth

**Affiliations:** 1 Cancer Research UK Beatson Institute, Switchback Road, Bearsden, Glasgow, G61 1BD, UK.; 2 Centre for Virus Research, University of Glasgow, Garscube Estate, Bearsden, Glasgow, G61 1QH, UK.; 3 School of Veterinary Medicine, University of Glasgow, Garscube Estate, Bearsden, Glasgow, G61 1QH, UK.; 4 Institute of Cancer Sciences, University of Glasgow, Garscube Estate, Bearsden, Glasgow, G61 1QH, UK.; 5 Department of Pathology, Western Infirmary, Glasgow, G11 6NT, UK.; 6 Goodman Cancer Research Centre, McGill University, Montreal, QC H3A 1A3, Canada.

**Keywords:** RUNX2, Breast cancer, Transgenic model, Mammary development

## Abstract

RUNX2, a master regulator of osteogenesis, is oncogenic in the lymphoid lineage; however, little is known about its role in epithelial cancers. Upregulation of *RUNX2* in cell lines correlates with increased invasiveness and the capacity to form osteolytic disease in models of breast and prostate cancer. However, most studies have analysed the effects of this gene in a limited number of cell lines and its role in primary breast cancer has not been resolved. Using a human tumour tissue microarray, we show that high RUNX2 expression is significantly associated with oestrogen receptor (ER)/progesterone receptor (PR)/HER2-negative breast cancers and that patients with high RUNX2 expression have a poorer survival rate than those with negative or low expression. We confirm *RUNX2* as a gene that has a potentially important functional role in triple-negative breast cancer. To investigate the role of this gene in breast cancer, we made a transgenic model in which *Runx2* is specifically expressed in murine mammary epithelium under the control of the mouse mammary tumour virus (MMTV) promoter. We show that ectopic *Runx2* perturbs normal development in pubertal and lactating animals, delaying ductal elongation and inhibiting lobular alveolar differentiation. We also show that the *Runx2* transgene elicits age-related, pre-neoplastic changes in the mammary epithelium of older transgenic animals, suggesting that elevated RUNX2 expression renders such tissue more susceptible to oncogenic changes and providing further evidence that this gene might have an important, context-dependent role in breast cancer.

## INTRODUCTION

The RUNX transcription factors are a closely related family of genes implicated in a number of cancers (Blyth et al., 2005). These genes (*RUNX1*, *RUNX2* and *RUNX3*) exhibit context-dependent oncogenic and tumour-suppressive properties through pertinent effects on cell growth and viability. *RUNX1* is a common target for chromosomal translocation and mutation in leukaemia, whereas RUNX3 function is reportedly lost in epithelial cancers (Blyth et al., 2005; Chuang and Ito, 2010; Mangan and Speck, 2011). In contrast to these putative tumour-suppressor properties, all three genes promote lymphoma development in mice (Cameron and Neil, 2004; Castilla et al., 2004; Stewart et al., 1997), a pro-oncogenic function confirmed for *Runx1* and *Runx2* using transgenic models (Blyth et al., 2009; Blyth et al., 2001; Vaillant et al., 1999). Although oncogenic in the lymphoid compartment, very little is known about the role of RUNX2 in epithelial cancers. Most studies have assessed its role in breast and prostate cancer cell lines (Blyth et al., 2010; Pratap et al., 2011; Shore, 2005). RUNX2 is upregulated in metastatic breast cancer cell lines (Barnes et al., 2003; Nagaraja et al., 2006) and its inhibition in MDA-MB-231 cells reduces invasive capacity *in vitro*, and decreases osteolytic disease and tumour growth *in vivo* (Barnes et al., 2004; Javed et al., 2005; Pratap et al., 2009). Overexpressing *Runx2* in cell lines increases invasiveness and induces a transformed phenotype in 3D culture models, where cells become more proliferative and less polarised (Pratap et al., 2009). RUNX2 also regulates genes associated with tumour cell migration, metastasis and angiogenesis, such as those encoding bone sialoprotein, osteopontin, matrix metalloproteinases and vascular endothelial growth factor (Pratap et al., 2005; Pratap et al., 2011). Furthermore, because RUNX2 is essential for osteogenesis, it has been suggested that association with cancers that preferentially metastasise to bone might be due to osteomimicry (Barnes et al., 2003). However, the direct impact of RUNX2 in epithelial lineages *in vivo* has never been tested.

To assess the role of RUNX2 in breast cancer we assessed expression in a human tissue microarray (TMA). We find that RUNX2 is overexpressed in a particular subset of breast cancers that are oestrogen receptor (ER)/progesterone receptor (PR)/HER2-negative, and that high expression of RUNX2 is associated with poorer patient survival. To model overexpression *in vivo* we generated a novel transgenic line directly assessing the role of RUNX2 in the context of primary mammary tissue. Transgenic expression perturbs normal development in mouse mammary gland, demonstrating that levels of *Runx2* must be tightly regulated for normal mammary development to occur. Furthermore, *Runx2* transgenic mice develop late-onset hyperplastic changes in the mammary gland, suggesting that overexpression of RUNX2 renders epithelial tissues more susceptible to pre-neoplastic changes.

**TRANSLATIONAL IMPACT**

**Clinical issue**

The RUNX2 transcription factor, known to be oncogenic in the T-cell lineage, is upregulated in human breast cancer cell lines and correlates with invasive properties in these cells. However, studies of this transcription factor have been limited to a small number of cell lines, and the role of RUNX2 as a putative oncogene in primary breast cancer has not been demonstrated *in vivo*. There is also a lack of information on the effect of RUNX2 expression on normal mammary epithelium. Triple-negative breast cancer is a subtype of breast cancer for which there is no good molecular biomarker or targeted therapeutic approach. Because this subgroup often correlates with poor prognosis, there is an urgent clinical need to identify underlying molecular mechanisms that would aid in treating individuals with this aggressive form of breast cancer.

**Results**

To explore the role of *RUNX2* in breast cancer, the authors assessed expression of the gene in a cohort of human breast cancers using a tissue microarray. They report that high expression of RUNX2 is limited to a small number of primary operable breast cancers. However, high RUNX2 expression specifically associates with the triple-negative subtype and correlates with poorer patient survival. To further validate the putative oncogenic properties of this gene *in vivo*, a transgenic mouse model was generated in which *Runx2* was targeted to the mammary epithelium. Transgenic *Runx2* predisposes primary tissue to late-onset pre-neoplastic changes, and supra-physiological expression perturbed normal mammary development and function.

**Implications and future directions**

This study demonstrates that RUNX2 expression significantly associates with triple-negative breast cancer. Whether RUNX2 is acting as a neutral biomarker for this poor prognostic subset or actively contributing to its more aggressive phenotype remains to be determined. In addition, this work also demonstrates for the first time *in vivo* that *Runx2*, a key regulator of bone development, could also have a functional role in epithelial tissue. The potential functional role of RUNX2 in normal mammary development awaits confirmation using a conditional-knockout model. Overall, the findings reported in this paper suggest a clinically relevant role for RUNX2 in breast cancer that is worthy of further investigation.

## RESULTS

### RUNX2 expression specifically correlates with ER/PR/HER2-negative breast cancer

**Fig. 1:**
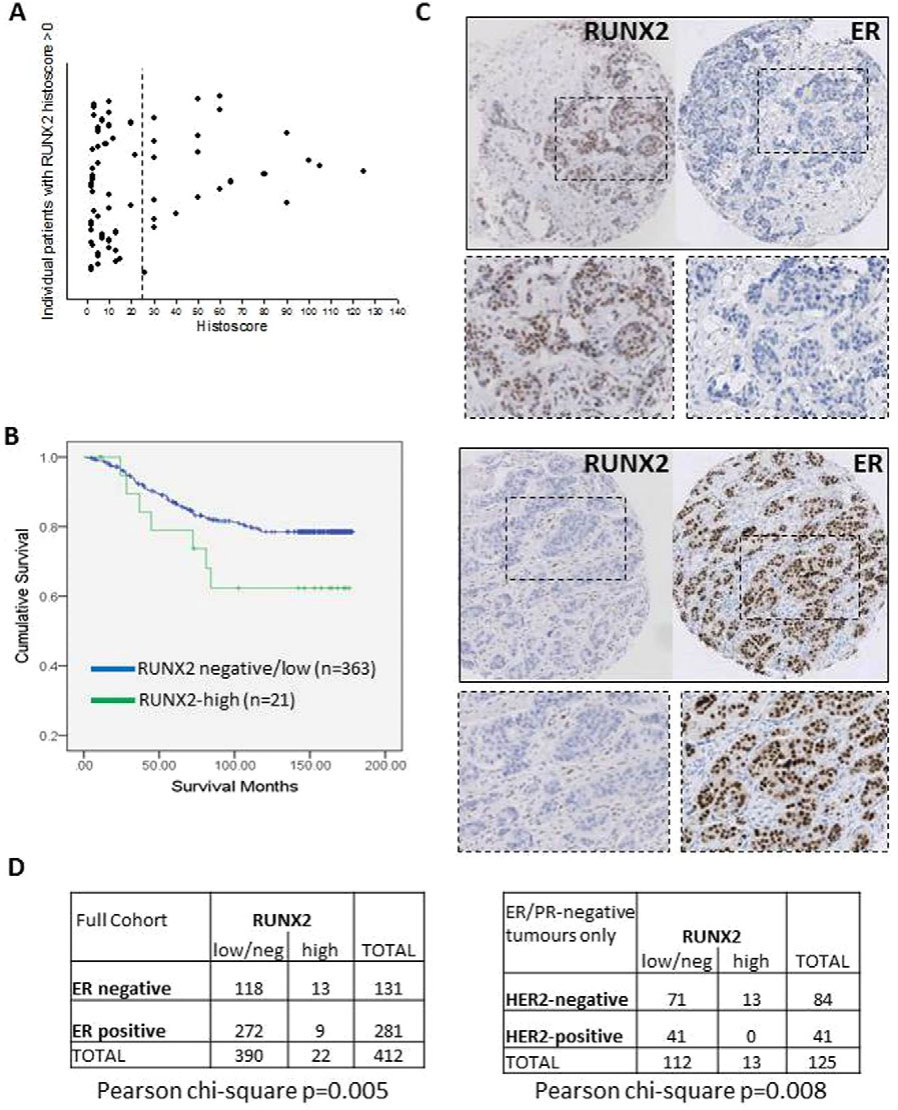
**Expression of RUNX2 correlates with ER/PR/HER2-negative human breast cancer.** Invasive breast carcinomas from a tumour tissue microarray (TMA-1) were stained for RUNX2. (A) Scatterplot showing the range of positive histoscores. Expression was divided into RUNX2-negative (histoscore 0), -low (histoscore 1–24) or -high (histoscore ≥25) in 416 breast cancers. The dotted line at histoscore 25 demonstrates the cut-off for RUNX2-high patients. Position on the *y*-axis reflects the order in which samples were analysed. (B) Kaplan-Meier of patient survival for 384 patients in A for which follow-up data was available. Survival is plotted for patients with high-RUNX2 tumours (*n*=21) and negative/low-RUNX2 tumours (*n*=363). (C) Examples of individual tumours stained for RUNX2 and ER, depicting the reciprocal expression pattern. Boxed areas are shown at higher magnification. (D) Significantly more RUNX2-high cancers were ER-negative (*P*=0.005; chi-square) and specifically associated with the triple-negative (ER/PR/HER2-negative) group (*P*=0.008; chi-square).

We assessed expression of RUNX2 in a cohort of human breast cancers using a tissue array (TMA-1). In samples of normal breast tissue examined, we detected negligible expression of RUNX2 with only occasional cells positive for the protein. Positive RUNX2 staining was detected in the epithelium of a proportion of tumours (67/416) and scored as negative in the remaining patients. Overall, RUNX2 expression was not associated with disease-specific survival, or with tumour grade, tumour size or lymph node status. Within the RUNX2-positive cohort there was a large group of patients displaying a low histoscore (Fig. 1A), raising concern over the functional significance of RUNX2 in these samples, especially in light of the fact that, in normal specimens, we observed occasional positive cells. We therefore applied a cut-off of weighted histoscore of 25 in the training set (TMA-1) and carried out Kaplan-Meier analysis (Fig. 1B), comparing tumours with high RUNX2 (histoscore >25) and negative/low RUNX2 (histoscore <25). Patients with breast cancers expressing high levels of RUNX2 had shorter overall survival (Fig. 1B; mean survival 130 months in RUNX2-high versus mean survival 152 months in RUNX2-low/negative). Owing to the small number of RUNX2-positive tumours, these results fail to reach statistical significance and warrant validation in a larger independent cohort. Importantly, RUNX2-expressing tumours were expressed at a significantly higher rate in ER-negative (13/131; 10%) compared with ER-positive (9/281; 3%) breast cancers (Fig. 1C,D; *P*=0.005; chi-square test). Furthermore, many (13/22) of the RUNX2-high tumours were negative for ER, PR and HER2 (the so-called triple-negative subtype). RUNX2-high tumours accounted for 13 of 84 (15%) triple-negative cancers in the cohort (Fig. 1D; *P*=0.008; chi-square test). High RUNX2 expression lowered mean survival of the triple-negative cohort from 137 to 119 months. We validated these results in a second independent TMA (TMA-2), using the same cut-off of weighted histoscore 25. In this cohort, which had an overall worse prognosis, high RUNX2 expression in ER/PR/HER2-negative tumours (7/38) was associated with a reduction in mean survival from 81 to 59 months. Assessment of clinicopathological characteristics showed that RUNX2-high tumours significantly associated with ER-negative (*P*<0.005) and PR-negative (*P*<0.002) status, and with rare breast cancer types (*P*<0.05; Table 1). There was no association with age, tumour size, Ki-67 or TUNEL, but there was a trend to more necrotic tumours (68% vs 53%) and with increased immune cell infiltrate (54.5% vs 37%; Table 1). Although these results will need testing in a larger cohort, it is tentative to say that RUNX2 could serve as a putative prognostic marker within the triple-negative subtype, which is valuable given the limited biomarkers and therapeutic treatment options available to this patient subset.

**Table 1:**
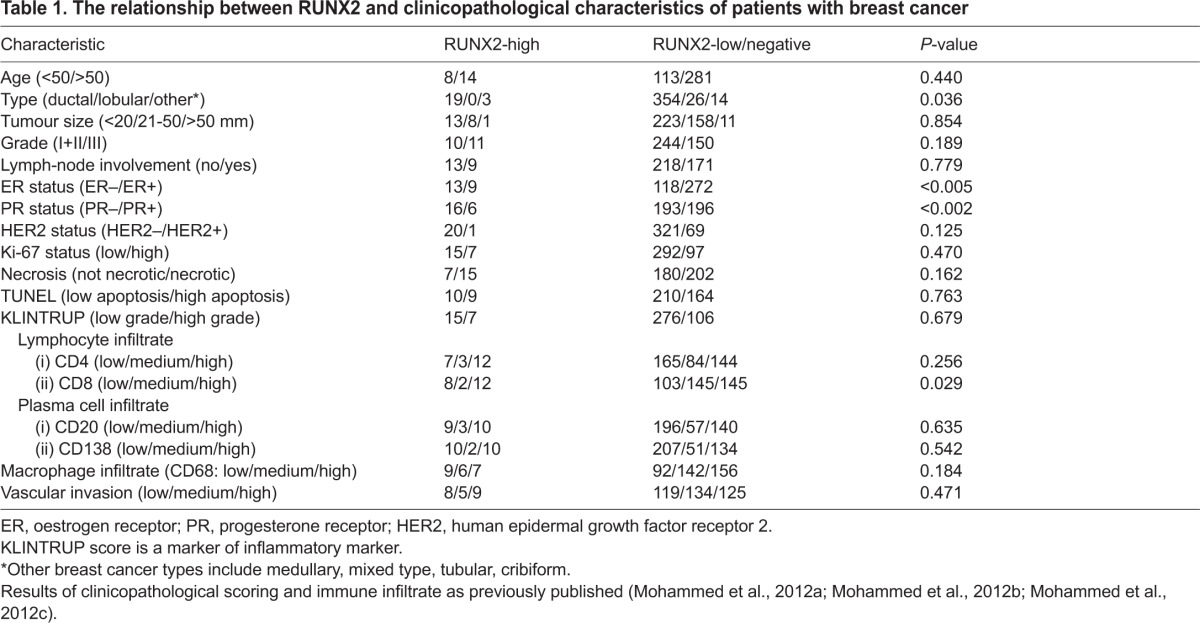
The relationship between RUNX2 and clinicopathological characteristics of patients with breast cancer

### Overexpression of *Runx2* perturbs development of virgin mammary glands in a transgenic model

**Fig. 2:**
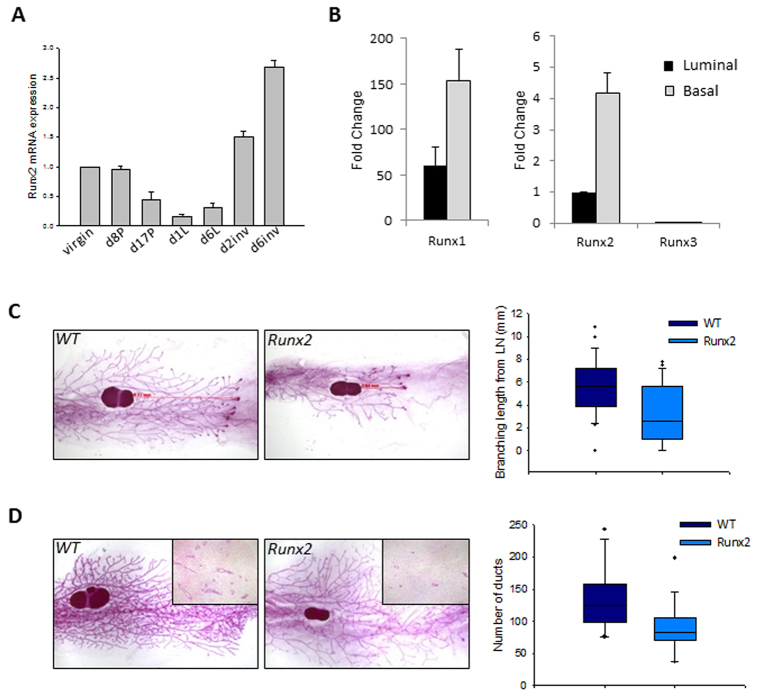
**Transgenic expression of *Runx2* perturbs pubertal mammary development.** (A) qRT-PCR of *Runx2* throughout murine mammary development. Expression levels relative to wild-type 12-week-old virgins; data are means ± s.d. (P, pregnant; L, lactating; inv, involution, d, day). (B) qRT-PCR of Runx expression in basal/myoepithelial and luminal epithelial cell populations sorted by FACS based on CD29 and CD24 surface markers (see text for details). *Runx1* is plotted on a different *y*-axis owing to the higher levels of expression. *Runx3* expression was not detectable in either population. Expression normalised to *Gapdh* is relative to luminal *Runx2*; data are means of three independent samples ± s.d. (C) Whole-mounts of 6-week-old mammary glands. Elongation from lymph node (LN) in weight- and litter-matched WT glands is greater than in MMTV-*Runx2* glands. Ductal elongation lengths as represented by the red arrows are quantified in the box plot (*P*=0.002; WT *n*=28; *Runx2 n*=23). Dots represent outliers. (D) Whole-mounts of 8-week-old MMTV-*Runx2* glands reveal a reduction in tertiary side-branching in *Runx2* glands compared with WT glands. Number of ducts per H&E section were counted for each sample (inset images); graph shows a significant reduction in average duct number in *Runx2* mammary glands (*P*=0.01; WT *n*=15; *Runx2 n*=16). Dots represent outliers. Whole-mounts, 6.5× magnification; H&Es, 40× magnification.

Despite being known to regulate specific mammary genes and being expressed in some breast cancer cell lines, very little is known about the normal function of RUNX2 in mammary gland development. To explore this we first examined endogenous *Runx2* expression in murine mammary gland during different physiological phases. *Runx2* is expressed in virgin, early pregnant and involuting glands, with levels decreasing during late pregnancy and lactation (Fig. 2A) (Blyth et al., 2010). To further dissect expression of *Runx2* in specific mammary cell types, we isolated basal/myoepithelial (lin^−^CD29^hi^CD24^+^) and luminal epithelial (lin^−^CD29^lo^CD24^+^) cells from wild-type (WT) pubertal mice on the basis of surface-marker expression and fluorescence-activated cell sorting (Shackleton et al., 2006). Expression of *Runx2* was fourfold greater in basal/myoepithelial cells compared with luminal epithelial cells (Fig. 2B). These results are consistent with studies from the Smalley lab where microarray and qRT-PCR analysis found *Runx2* in sorted basal/myoepithelial populations (Kendrick et al., 2008; Molyneux et al., 2010). *Runx1* was also higher in this population and, in agreement with what we (Blyth et al., 2010) and others (Wang et al., 2011) have shown, *Runx1* is the most highly expressed of the Runx genes in mammary epithelial cells. *Runx3* was not detected in sorted mammary epithelial cells (Fig. 2B).

Our TMA results indicated that high-level RUNX2 expression could be a marker for a subset of cancers with poor prognosis. To investigate the effects of deregulated expression, we generated a novel transgenic model by targeting *Runx2* to mammary epithelium using the mouse mammary tumour virus (MMTV) promoter (MMTV-*Runx2*). Germline transmission was achieved in two founder lines, and transgene expression confirmed (supplementary material Fig. S1). Development of the murine mammary gland begins after birth, when terminal end bud structures migrate through the fat pad until the entire gland is filled with an epithelial structure. This process was perturbed in the *Runx2* transgenic mice. When comparing MMTV-*Runx2* females to weight-matched 6-week-old WT mice, there was a delay in ductal elongation, with a significant reduction in the distance that epithelial structures extend through the fat pad (Fig. 2C; WT *n*=28, Runx2 *n*=23, *P*=0.002). By 8 weeks of age, MMTV-*Runx2* females overcame this delayed elongation phenotype but, at this stage, a reduction in tertiary side-branching was evident (Fig. 2D). Quantification by counting the number of ducts in histological sections showed that MMTV-*Runx2* females displayed over 30% fewer ducts than controls (Fig. 2D; WT *n*=15, Runx2 *n*=16, *P*=0.01). Hormone signalling plays a crucial role in mammary gland development; however, ectopic *Runx2* expression does not significantly alter the number of PR+ or ERα+ cells (supplementary material Fig. S2). Neither does the observed phenotype result from changes in lineage specification, because transgenic and control animals have comparable proportions of basal and luminal cells, as well as CK5 and CK18 populations (supplementary material Fig. S3). Transgenic *Runx2* expression therefore perturbs normal development with delayed elongation and reduced tertiary branching of the mammary epithelial tree in young virgin females.

### Ectopic *Runx2* expression delays lobular alveolar differentiation, resulting in a lactation defect

**Fig. 3:**
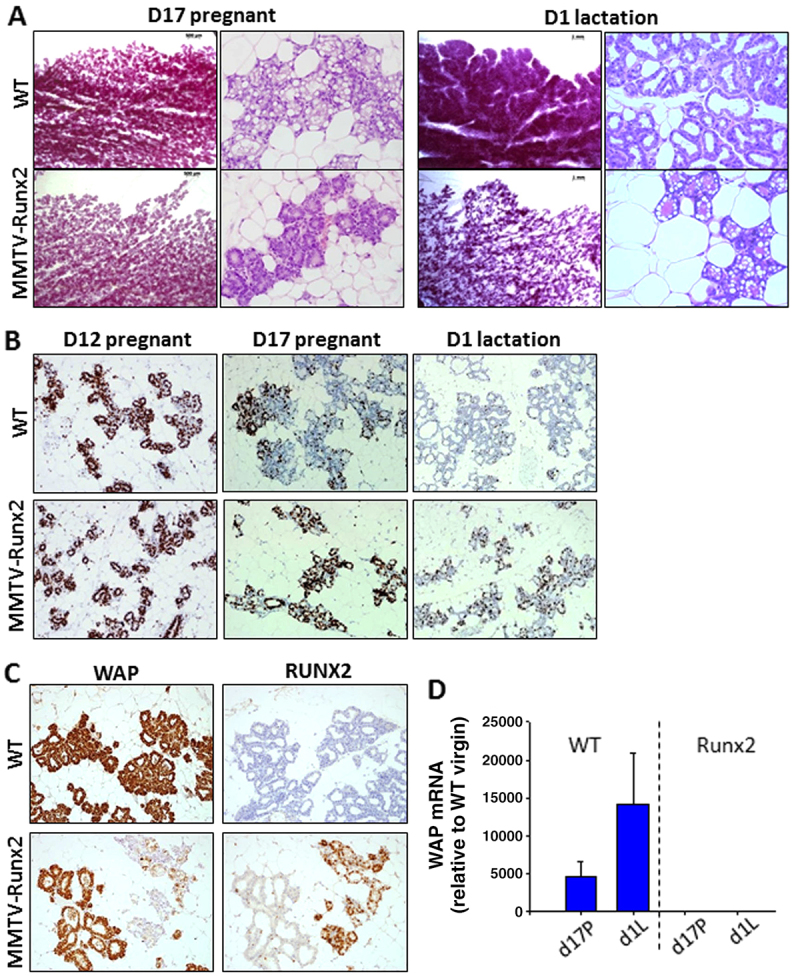
**Ectopic RUNX2 expression leads to delayed differentiation and a lactation defect.** (A) Whole-mount and histological analysis of MMTV-*Runx2* glands (*n*=11) during late pregnancy (D17 pregnant) reveals a reduction in side-branching and alveolar expansion compared with WT (*n*=14), and failure to form mature alveolar units during lactation (WT *n*=13; *Runx2 n*=11). (B) Ki67 staining of WT and MMTV-*Runx2* glands at day 12 (D12) pregnancy (*n*=4 each), late pregnancy (D17P; *n*=6 each) and day 1 of lactation (D1; WT *n*=6; *Runx2 n*=7). (C) Immunohistochemistry of whey acidic protein (WAP) and RUNX2 on serial sections at day 1 lactation in WT (*n*=6) and MMTV-*Runx2* (*n*=7) glands illustrates their reciprocal expression pattern. (D) Quantification of *Wap* mRNA with dramatically less *Wap* in *Runx2* glands at late pregnancy (d17P) and lactation (d1L); data are means ± s.d. normalised to HPRT relative to WT virgin. Whole-mounts, 8× magnification; H&Es, 200× magnification; IHC images, 100× magnification in B and 200× magnification in C.

Although MMTV-*Runx2* transgenic females developed normally, were fertile and had normal litter sizes, mothers consistently failed to nurse their pups, which then died with little or no milk in their stomachs. WT and transgenic glands appeared phenotypically similar at mid-pregnancy, with comparable levels of proliferation (D12, Fig. 3B). However, defects in mammary gland architecture were observed by late pregnancy, with reduced ductal side-branching and alveolar expansion, and a failure of terminal differentiation resulting in a significant reduction in mature alveolar units (Fig. 3A). There was no difference in apoptosis of glands as assessed by caspase-3; however, inappropriate cell cycling (Fig. 3B) was seen in what should be a fully differentiated organ at lactation, exemplifying the delayed development of the post-parturient gland. The differentiation marker whey acidic protein (WAP; a constitutive milk protein) was detected at much lower levels compared with in WT (Fig. 3C,D) and was absent from areas showing high transgene expression (Fig. 3C), demonstrating a block in terminal alveolar differentiation.

**Fig. 4:**
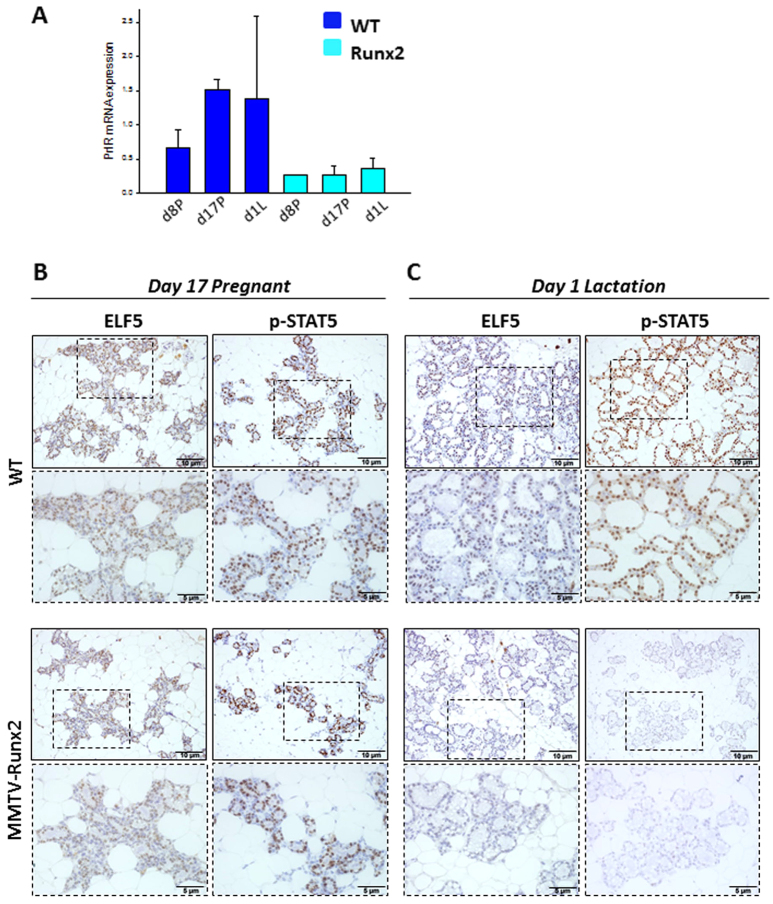
**Ectopic *Runx2* inhibits ELF5 and p-STAT5 during lactation.** (A) Prolactin receptor (*Prlr*) mRNA expression at pregnancy (d8P, d17P) and lactation (d1L) in *Runx2* and WT glands; data are means ± s.d., normalised to HPRT and relative to WT virgin. (B) ELF5 and p-STAT5 protein levels are not affected in MMTV-*Runx2* glands at late pregnancy (day 17) but, during lactation, *Runx2* overexpression causes a reduction in levels of ELF5 and p-STAT5 (C). Each image is representative of *n*≥3 mice. Boxed areas are shown at higher magnification. Scale bars: 10 μm (5 μm in higher-magnification images).

Prolactin hormone activity is crucial for terminal differentiation, which leads to normal milk-producing alveolar units during pregnancy and lactation. Prolactin receptor (*Prlr*) expression was reduced in pregnant and lactating MMTV-*Runx2* glands (Fig. 4A). Expression of proteins downstream of PRLR, namely ELF5 and p-STAT5, were unaffected by RUNX2 overexpression during late pregnancy (Fig. 4B). However, at the onset of lactation, ELF5 and p-STAT5 levels were reduced in MMTV-*Runx2* glands (Fig. 4C). This is an important observation; during late pregnancy, *Runx2*-expressing tissue, although abnormally differentiated, was still able to express factors crucial for normal alveolar development. However, at lactation, *Runx2* expression seems to inhibit ELF5 and p-STAT5 expression, blocking their normal function of driving alveologenesis. We also assessed GATA3 in the lactating transgenic glands but saw no difference compared with the controls (data not shown).

**Fig. 5:**
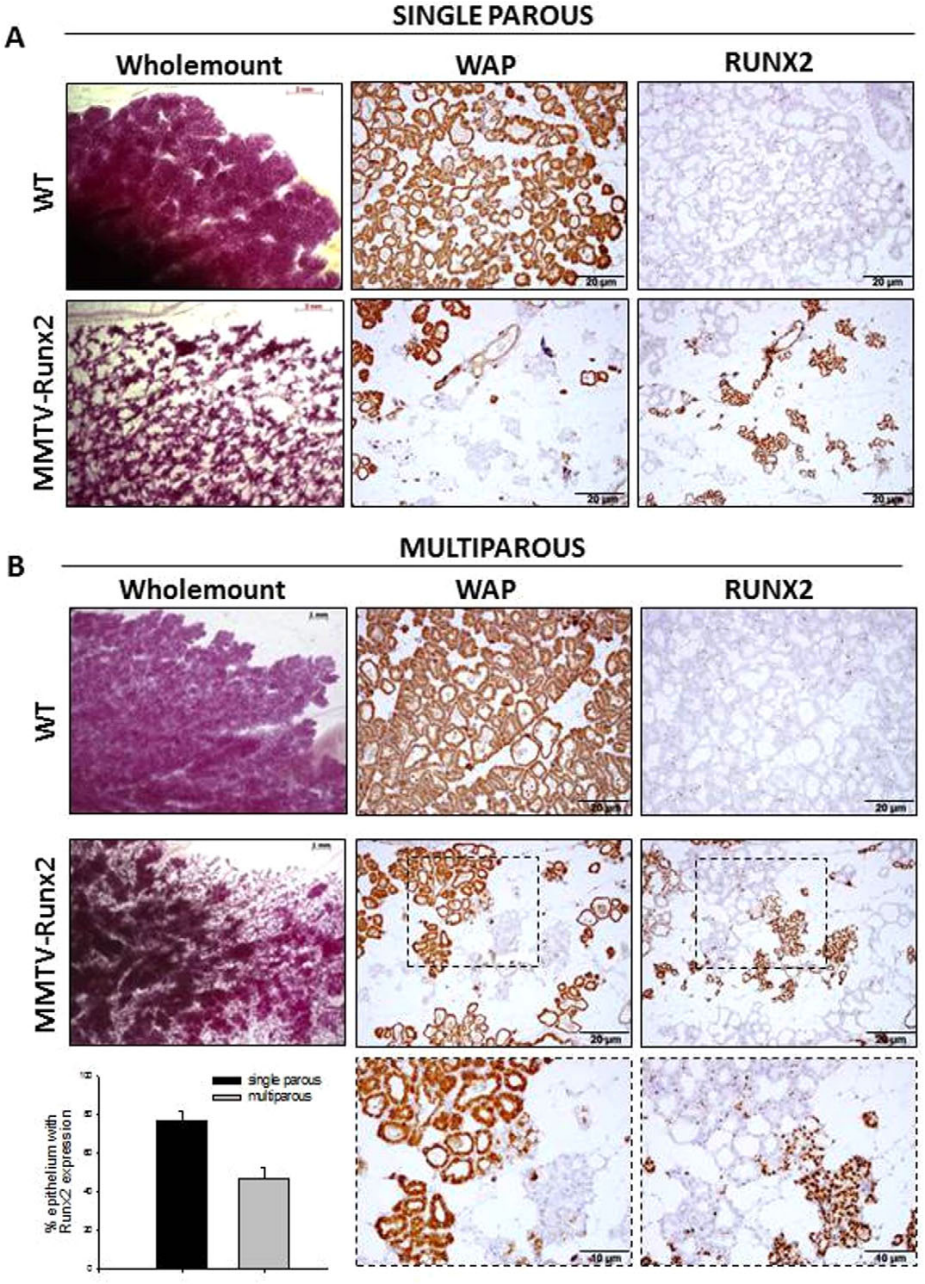
***Runx2*-induced lactation failure is partially rescued by multiple parities.** Transgenic females were housed with co-fostered WT females to achieve continued suckling from pups. Representative images for single parous (A) and multiparous (≥4 pregnancy) (B) co-fostered females at day 1 lactation. Whole-mounts show the retarded development in transgenics, although *Runx2* multiparous glands have more mature alveolar formation, with glands evidently fuller and producing more WAP (B) than *Runx2* single-parity females (A). Multiparous transgenic glands proportionally express less RUNX2 than single-parity glands, which is reciprocal to the higher levels of WAP as shown in serial sections in A and B. Boxed areas are shown at higher magnification. Single-parous females: WT *n*=6, *Runx2 n*=7; multiparous females: WT *n*=3, *Runx2 n*=3. Quantification of RUNX2 positivity per total epithelium in multiparous (grey) and single-parous (black) glands is given in the bar graph (*P*=0.0001; single parous *n*=5; multiparous *n*=3). Scale bars in wholemounts: 2 mm in A and 1 mm in B; scale bars in IHC images: 20 μm (10 μm in higher-magnification images).

This lactation defect could not be rescued by co-fostering, in which continued suckling activity is achieved in the presence of a lactating foster mother (Fig. 5A). However, transgenic females with multiple pregnancies (≥4) could rear small litters. Multiparous *Runx2* glands had more mature alveolar units, reduced RUNX2 expression and a reciprocal rise in WAP expression compared with single-parous *Runx2* females (Fig. 5). Interestingly, areas of multiparous glands that expressed the transgene still showed delayed development and a reduction in ELF5 and p-STAT5 (supplementary material Fig. S4). Therefore, ectopic *Runx2* elicits a lactation defect by inhibiting terminal differentiation possibly by suppressing the normal function of the PRLR pathway.

### Mammary glands of aged MMTV-*Runx2* females display pre-neoplastic changes

**Fig. 6:**
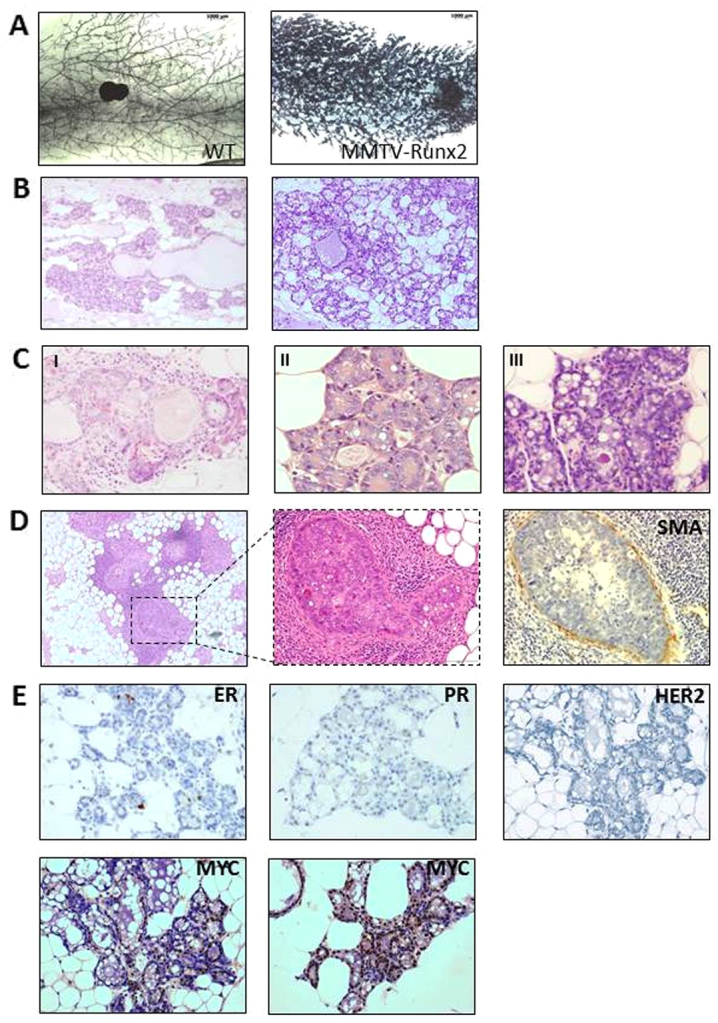
**Mammary glands of aged MMTV-*Runx2* females display abnormal hyperplastic and pre-neoplastic changes.** (A) Representative whole-mounts of aged MMTV-*Runx2* and WT littermate controls (8× magnification). (B) Representative images of H&E sections showing diffuse hyperplasia in two independent MMTV-*Runx2* transgenic glands with evidence of secretory hyperplastic lesions and dilated ducts (100× magnification). (C) Abnormal features observed in aged MMTV-*Runx2* glands, such as distorted acini with lobular fibrosis and chronic inflammatory cell infiltrate (I), and alveolar hyperplasia with luminal cells exhibiting large nuclei and prominent nucleoli (II,III) (images shown at 400× magnification). (D) Ductal carcinoma *in situ* (DCIS) in an MMTV-*Runx2* female; middle panel is higher magnification of boxed area. Smooth muscle actin (SMA) staining shows an intact basal/myoepithelial layer. (E) Hyperplastic lesions are negative for ER, PR and HER2 but show positivity for MYC as determined by immunohistochemistry (400× magnification).

Although we observed developmental perturbation, the ultimate aim of our model was to explore the potential oncogenic role of *Runx2* in mammary epithelium. Transgene expression in aged females produced a dramatic and significant (*P*<0.001) expansion of epithelial tissue with atypical hyperplastic and pre-neoplastic lesions. Of 47 MMTV-*Runx2* females aged between 16 and 24 months, 26 (55%) displayed alveolar expansion with focal or diffuse hyperplasia and dilated ducts filled with secretory material (Fig. 6A,B). Only 5/33 (15%) wild-type controls exhibited similar characteristics. This phenotype was observed in both virgin (*n*=18/31; 58%) and parturient (*n*=8/16; 50%) transgenic females; therefore, hyperplasia was not a consequence of abnormal involution and/or clearing of alveolar tissue. Expanded epithelial tissue often manifested as secretory hyperplastic areas (Fig. 6B) with abnormal features such as distorted acini with lobular fibrosis, chronic inflammatory cell infiltrate and focal squamous metaplasia (Fig. 6C–I), and as alveolar hyperplasia with luminal cells exhibiting large nuclei and prominent nucleoli (Fig. 6C-II,C-III). A low incidence of multifocal ductal carcinoma *in situ* (DCIS, *n*=3/47; 6%) was observed in MMTV-*Runx2* females (Fig. 6D), whereas no DCIS were seen in control glands (0/33). SMA staining showed that these were non-invasive lesions (Fig. 6D; *n*=3). Prevalent hyperplastic lesions were negative for ER, PR (Fig. 6E; *n*=11) and HER2 (Fig. 6E; *n*=4). Interestingly, lesions were positive for MYC (Fig. 6E; *n*=4), which has been shown previously to act cooperatively with RUNX2 to drive T-cell lymphoma (Blyth et al., 2006). Therefore, ectopic *Runx2* elicits long-term remodelling of the mammary gland promoting development of pre-neoplastic changes in the epithelium.

## DISCUSSION

*Runx2* exerts a dominant oncogenic role in T-cell lymphomas (Blyth et al., 2001; Blyth et al., 2006); however, little evidence about its role in primary epithelial cancers has been described. Work on well-characterised breast cancer cell lines has shown that RUNX2 can influence the behaviour of these cells and the response of host tissues following orthotopic injection to bone (Barnes et al., 2004; Javed et al., 2005; Pratap et al., 2008), but this work has been limited to a few cell lines. We now describe the first *in vivo* study of the pro-oncogenic effects of RUNX2 in breast epithelium.

High RUNX2 expression is limited to a subpopulation of breast cancer: it significantly associates with triple-negative (ER/PR/HER2-negative) disease and correlates with poorer patient survival. Its potential as a putative prognostic marker in this subtype is tantalising but, owing to the small number of positive samples, requires testing in a larger cohort. Notably, expression of RUNX2 is specifically observed in cell lines of the ‘basal-like’ subtype (ER/PR/HER2-negative) and not in those derived from ‘luminal-like’ tumours (Lau et al., 2006). Moreover, MMTV-*Runx2* failed to promote tumorigenesis in MMTV-*PyMT* and MMTV-*ErbB2* models (not shown), which have been molecularly compared to luminal (ER+) breast cancers (Herschkowitz et al., 2007), again highlighting its role in subtype-specific disease. The Frenkel group (Chimge et al., 2011; Khalid et al., 2008) also showed that expression of RUNX2 and its target genes inversely correlated with ER expression, whereas overexpression of ER in MDA-MB-231 cells inhibited RUNX2. Although these results are concordant with a small study showing that RUNX2 is absent in ER+ breast cancer (Onodera et al., 2010), they contrast to one in which RUNX2 expression was specifically found in ER+ tumours (Das et al., 2009). Interestingly, RUNX2 has never been identified in transcriptomic analyses of breast cancer, but this might be due to problems with RUNX2 probe sets, and, using a RUNX2 metagene as a surrogate for RUNX2 activity, it has been shown that high expression of RUNX2-target genes correlates with basal-like breast cancers (Chimge and Frenkel, 2013), in support of our own conclusions.

A mammary-specific *Runx2* transgenic model was generated to definitively test its role in primary epithelium. The most striking phenotype of MMTV-*Runx2* mice was extensive changes characterised by hyperplasia, dysplasia and lesions consistent with a pre-neoplastic phenotype in aged females, observed in both virgin and parturient females. Some of the changes, even in virgin animals, resemble the secretory phenotype of a pregnant gland, suggesting attenuation of control mechanisms that keep mammary stem or progenitor cells quiescent, ultimately disrupting epithelial homeostasis. Such an effect could be intrinsic to the epithelial compartment or relate to how persistent *Runx2* expression affects the mammary environment. RUNX2 regulates the expression of extracellular matrix proteins (Komori, 2010) and cross-talk between epithelium and stroma is important for development and progression of breast cancer (Place et al., 2011). Our results reveal the potential of *Runx2* to exert oncogenic effects in primary epithelium; however, the lack of overt carcinoma in mice suggests that additional genetic mutations are required. Indeed we note high levels of MYC in the lesions, which has been shown previously to be a strong collaborating event with RUNX2 in lymphoid tumours (Blyth et al., 2001; Blyth et al., 2006). Alternatively, because RUNX2 expression correlates with basal-like human disease, it could be that the MMTV-driven expression in the transgenic model does not effectively target the most susceptible cell population. Interestingly, the hyperplastic lesions were negative for ER, PR and HER2 expression, which might be related to the role of RUNX2 in antagonising ER signalling (Chimge et al., 2012; Chimge and Frenkel, 2013; Khalid et al., 2008).

Expression of the Runx genes is tightly controlled in mammary epithelia, both spatially and temporally. In keeping with this, RUNX2 regulates a number of genes involved in mammary function (Inman et al., 2005; Inman and Shore, 2003). Cells of the basal/myoepithelial lineage express significantly higher levels of *Runx1* and *Runx2* than luminal cells. Because this population harbours stem cells capable of generating whole glands from a single cell (Shackleton et al., 2006), it is tempting to speculate that Runx genes might regulate mammary stem cell or progenitor function (Ferrari et al., 2013), especially because Runx genes are already known to have a homeostatic role in haematopoietic stem cells (Swiers et al., 2010). Expression was higher in the virgin gland and fell during pregnancy and lactation. Consistent with this functional pattern of expression, ectopic *Runx2* prevented terminal differentiation of mammary cells in late pregnancy and inhibited development of alveoli, with resultant agalactia. Interestingly, multiple pregnancies can partially rescue the lactation defect. Gil Smith and colleagues have described the existence of mammary progenitor cells that persist in the mammary gland after involution and can contribute to the formation of alveolar units at subsequent pregnancies (Boulanger et al., 2005; Wagner et al., 2002). Given that the *Runx2* transgene shows a mosaic pattern of expression, one possible explanation is that parity-induced progenitor cells expressing the transgene have a selective disadvantage and are outcompeted over multiple pregnancies, with ultimate recovery of function.

The phenotype we observed in MMTV-*Runx2* lactating females is reminiscent of knockout models downstream of PRLR signalling, such as *Elf5* and *Stat5*. PRLR signalling is essential for terminal differentiation and lactation function, and mammary-specific deletion models of *Elf5* and *Stat5* display abnormal alveologenesis and lactation defects (Choi et al., 2009; Liu et al., 1997; Oakes et al., 2008). It is of note that there is a reduction in ELF5 and p-STAT5 in *Runx2*-expressing glands with aberrant alveologenesis. Under normal circumstances, *Runx2* might be suppressed to allow proper PRLR signalling and lactation, whereas, in the overexpression model, *Runx2* inhibits the PRLR cascade, culminating in a lactation deficiency.

Thus, ectopic expression of *Runx2* perturbs development and differentiation in the mouse mammary gland, suggesting that levels must be tightly controlled for normal development. Transgenic expression also predisposes to precancerous lesions, showing for the first time a pro-oncogenic role in mammary epithelium. In this regard, RUNX2 is significantly associated with triple-negative breast cancer and patients with higher RUNX2 expression have poorer survival rates. Whether RUNX2 is acting as a neutral biomarker for this poor prognostic subset or actively contributing to a more aggressive phenotype remains to be determined but is worthy of further investigation with important clinical relevance.

## MATERIALS AND METHODS

### Transgenic mice

To generate MMTV-*Runx2* mice, murine p1 *Runx2* polyA cassette of Cbfa1-G1 (Vaillant et al., 1999) was excised by *Eco*RV/*Xba*I digestion and cloned into a 5.63-kb *Bmg*BI-*Xba*I fragment of MMTV construct (kindly supplied by Alexander Borowsky, UC Davis Comprehensive Cancer Center, Sacramento, CA) (supplementary material Fig. S1A). pBluescript sequences were removed by *Xho*I/*Sac*II digestion. MMTV-Runx2p1 polyA insert was purified, and linearised DNA microinjected into C57Bl/6 × CBA/Ca F2 embryos. All animal work was carried out under the Animal (Scientific Procedures) Act 1986 and the EU Directive 2010 (PPL 60/4181).

### Wholemount/histological analysis of mammary glands

Inguinal glands were dissected, fixed in Carnoy’s, rehydrated through ethanol and stained with carmine alum or haematoxylin. Glands were dehydrated, cleared in xylene, mounted in Permount (Thermo Fisher) and captured with Zeiss stereomicroscope. For histological analysis, glands were dissected into neutral buffered formalin and H&E stained.

### Immunohistochemistry

Paraffin-embedded tissue sections were rehydrated before antigen retrieval using pH 6 sodium citrate buffer (or 1 mM EDTA pH 8 for WAP). After washing with Tris-buffered saline and blocking endogenous peroxidase, sections were incubated for 1 hour at room temperature (RT) with the following HRP-conjugated primary antibodies: anti-RUNX2 (Sigma HPA022040; 1/100), anti-cytokeratin-5 (Covance PRB-160P; 1/500), anti-cytokeratin-8/18 (Fitzgerald 20R-CP004; 1/500 overnight at 4°C), anti-ERα (Santa Cruz sc-542; 1/250), PR (Santa Cruz sc-539; 1/250), anti-Ki67 (Thermo Fisher RM-9106-S1; 1/250), anti-ELF5 (Santa Cruz sc-9645; 1/100), anti-p-STAT5 (Cell Signaling 9359S; 1/300), anti-SMA (Sigma A2547; 1/6000), anti-c-MYC (Santa Cruz sc-764; 1/200), anti-HER2 (Cell Signaling CS#2165; 1/400) and anti-WAP (Santa Cruz sc-14832; 1/2500). Additional anti-RUNX2 antibodies were used for validation in human samples (R&D Systems AF2006; Santa Cruz sc-10758x; MBL D130-3). Anti-rabbit secondary (Dako EnVision) was used for all antibodies except CK8/18 (anti-guinea pig; Sigma A9167, 1/600), SMA (Dako EnVision mouse kit) and WAP/ELF5 (anti-goat; Impress goat kit). Sections were incubated with secondary antibodies for 50 minutes at RT, treated with DAB and counterstained with haematoxylin.

### Quantitative RT-PCR

SYBR-Green-based quantitative PCR was carried out and data analysed using MJ Chromo4 (Bio-Rad). Primers: Runx1 (Qiagen Quantitect QT0010000380), Runx2 (Qiagen Quantitect QT00102193), Runx3-Fwd (5′-GCACCGGCAGAAGATAGAAGAC-3′), Runx3-Rev (5′-GGTTTAAGAAGCCTTGGATTGG-3′), PrlR (Qiagen Quantitect QT00154154), WAP-Fwd (5′-TTGAGGGCACAGAGTGTATC-3′), WAP-Rev (5′-TTTGCGGGTCCTACCACAG-3′), HPRT (Qiagen Quantitect QT00166768) and GAPDH (PrimerDesign kit). All reactions were performed in triplicate, and expression normalised to HPRT/GAPDH.

### Western blot

Nuclear extracts were prepared from tissue using NE-PER Nuclear and Cytoplasmic Extraction Reagents (Thermo Scientific). 20 μg extract was resolved on 10% NuPAGE Novex Bis-Tris gels (Invitrogen) and transferred to Hybond-ECL nitrocellulose membranes (Amersham). Membranes were probed with RUNX2 (Sigma), ERK (Cell Signaling) and GAPDH (Cell Signaling).

### Cell lines

Epithelial cells were extracted from pregnant mammary glands for RUNX2 western. *Runx2*^−/−^ mouse embryonic fibroblasts (Rx2KO MEFs) (Kilbey et al., 2007) were used as negative control. MDA-MB-231 was purchased from American Type Culture Collection. MDA-MB-231 cells were transfected with *RUNX2* shRNA and scrambled control (HuSHTM, Origene) through electroporation using Nucleofector Kit V program X-013 (Amaxa, Lonza). For immunohistochemistry, cells were fixed with 2% paraformaldehyde for 15 minutes. After centrifugation, pellets were resuspended in 200 μl of 3% UltraPure^™^ low-melting agarose (Invitrogen) and left for 20 minutes at RT to solidify. The agarose plug in 70% ethanol was embedded in paraffin blocks and stained as above.

### Flow cytometry/cell sorting

Mammary glands were dissected from 12-week-old virgin females (*n*≥3) to obtain epithelial cells, and labelled with CD24-PE, CD29-FITC, CD31-APC and CD45-APC (BD Biosciences) for flow cytometry (Shackleton et al., 2006). Live *Lin^−^* cells (DAPI/CD31/CD45-negative) were gated on FACSAria (BD Biosciences) for CD24^hi^CD29^lo^ (luminal) and CD24^hi^CD29^hi^ (basal/myoepithelial) cells using FlowJo software. For cell sorting, these populations were collected and processed for qRT-PCR.

### Human tissue microarray

Expression studies in human tissues were ethically approved [LREC Ref: Project Number 02/63(1) R&D project: 02PA002 and REC Ref: Project Number 02/SG007(10), R&D project: RN07PA001]. Tissue microarrays were already available. 0.6 mm^2^ breast cancer tissue cores, identified by a pathologist (Dr Elizabeth Mallon, Southern General Hospital), were removed from representative areas of tumours taken from patients at surgical resection. Tissue microarray blocks were constructed in triplicate. All patients in both the training set (TMA-1) and validation cohorts (TMA-2) were selected from a retrospective audit of clinical characteristics of 1743 patients diagnosed with operable invasive breast carcinoma between 1980 and 1999 in the Greater Glasgow and Clyde area, where clinical specimens were available. Cohort characteristics were similar (Table 2).

**Table 2:**
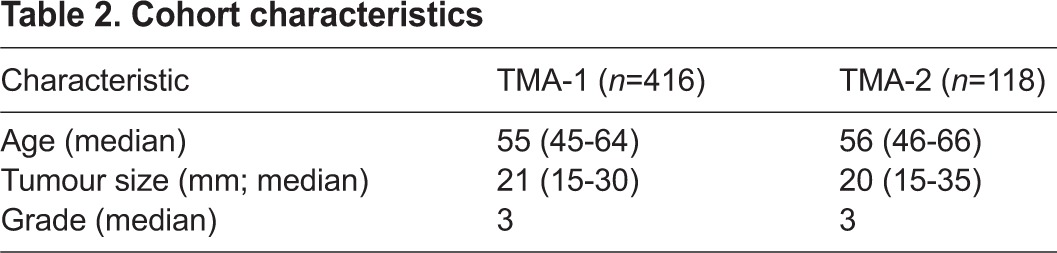
Cohort characteristics

Patients received standard adjuvant treatment according to protocols. Follow-up details included information on clinical attendances, recurrence and metastasis, date and cause of death as well as adjuvant therapy details. ER, PR (Mohammed et al., 2012a) and HER2 status (Mohammed et al., 2012c), and immune and inflammatory infiltrate (Mohammed et al., 2012b) were already available. Tissue work was conducted with strict adherence to REMARK Criteria (Altman et al., 2012), where inclusion criteria was availability of clinical information and tissue for analysis, and exclusion criteria was missing data and tissue. Because this was a retrospective cohort, treatment was chosen by the health care team according to what was most appropriate for the patient at the time. Tissue microarrays were stained for RUNX2 by immunohistochemistry. Anti-RUNX2 antibody validation was confirmed in MDA-MB-231 cells with shRUNX2 knockdown stained using the same protocol as the TMA, and by western blot (supplementary material Fig. S5). RUNX2 was quantified using weighted histoscore method to give a value of 0–300 (Kirkegaard et al., 2006). 230 cores (15% of total core number) were scored independently for RUNX2 by two observers (L.M. and N.F.) who were blind to patient’s outcome and each other’s score. Agreement between observers, calculated using interclass correlation coefficient (ICCC), was 0.84, classed as very good. L.M. then scored all cores and this data was used in the analysis.

### Statistical analysis

Statistical significance (*P*<0.05) of differential findings between experimental groups was determined by Student’s *t*-test using SigmaPlot 8.0. For human studies, SPPS19 (Chicago) was used and disease-specific survival rates generated using Kaplan-Meier. Log-rank test compared significant differences between subgroups using univariate analysis. Interrelationships between clinical parameters, PR and HER2 status, were calculated using chi-square test. Missing data was not boot-strapped and therefore not considered in the analysis.

## Supplementary Material

Supplementary Material
