# Supplementary material for: RUNX2 correlates with subtype-specific breast cancer in a human tissue microarray, and ectopic expression of Runx2 perturbs differentiation in the mouse mammary gland
Source: Dis Model Mech. 2014 Mar 13;7(5):525–34. doi: 10.1242/dmm.015040 (PMC4007404; doi:10.1242/dmm.015040)
Supplement: Supplementary Material [file supp_7_5_525__index.html]

RUNX2 correlates with subtype-specific breast cancer in a human tissue microarray, and ectopic expression of Runx2 perturbs differentiation in the mouse mammary gland — Supplementary Material 

# RUNX2 correlates with subtype-specific breast cancer in a human tissue microarray, and ectopic expression of *Runx2* perturbs differentiation in the mouse mammary gland

## DMM015040 Supplementary Material

**Files in this Data Supplement:**

- **Supplementary Material**
