# Supplementary material for: RUNX2 correlates with subtype-specific breast cancer in a human tissue microarray, and ectopic expression of Runx2 perturbs differentiation in the mouse mammary gland
Source: Dis Model Mech. 2014 Mar 13;7(5):525–34. doi: 10.1242/dmm.015040 (PMC4007404; doi:10.1242/dmm.015040)
Supplement: Supplementary Material [file supp_7.5.525_DMM015040.pdf]

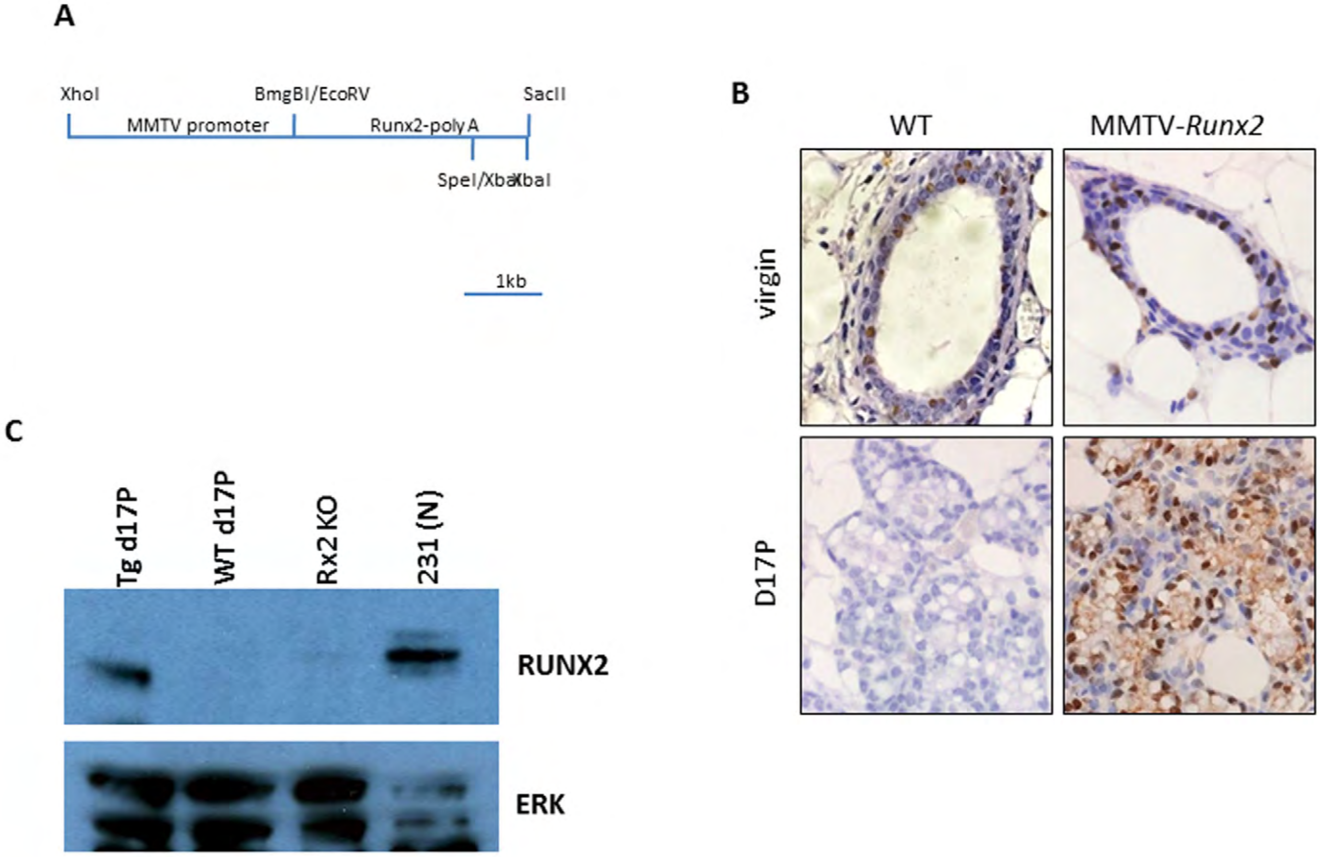

Supplementary Figure S1. Expression of *Runx2* in transgenic mammary glands. (A) MMTV-*Runx2* construct showing insertion of *Runx2* polyA cassette (containing the entire coding sequence of murine *Runx2* P1 isoform) into pBluescript MMTV vector. (B) RUNX2 immunohistochemistry; virgin and late pregnant (D17P) mammary glands of wild-type (WT) and transgenic (MMTV-*Runx2*) females (x40). (C) Western blot (nuclear extracts) confirms increased RUNX2 expression in transgenic (Tg) versus wild-type (WT) d17 pregnant glands. MDA-MB-231 (231) and Runx2-knockout (Rx2KO) MEFs as positive and negative controls respectively. Total ERK as a loading control.

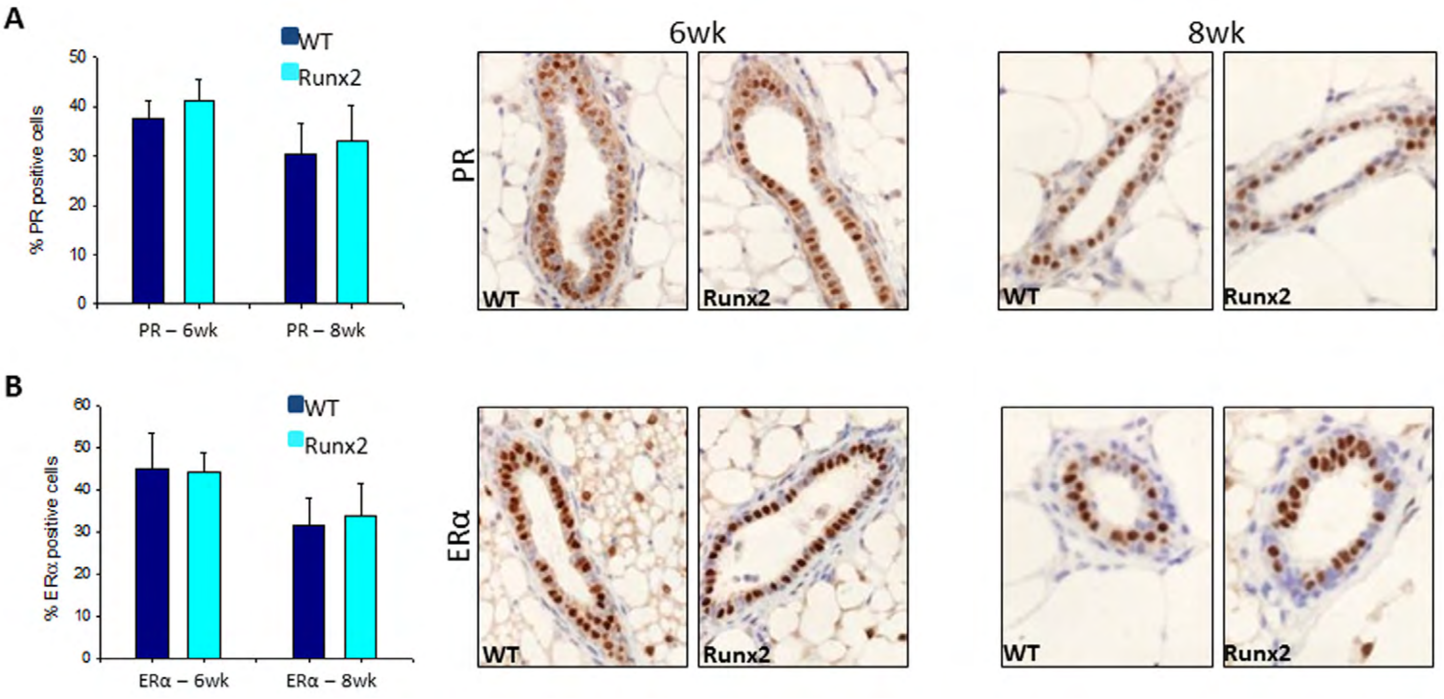

Supplementary Figure S2. Ectopic *Runx2* expression does not affect ERα/PR cell distribution. (A) The number of progesterone receptor (PR) cells is not significantly altered in MMTV-*Runx2* glands in 6wk (p=0.07; WT n=11 V *Runx2* n=9) or 8wk old (p=0.56; WT n=5 V *Runx2* n=5) virgin glands. Data are means ±SD. (B) The number of ERα cells was comparable between WT and *Runx2* virgin glands at 6wks (p=0.92; WT n=6 V *Runx2* n=5) and 8wks (p=0.25; WT n=5 V *Runx2* n=6). Data are means ±SD. For PR/ERα scoring, total cell number was counted at x40 magnification in 10 ducts per sample; PR/ERα positive cells counted and shown as percentage of total cells.

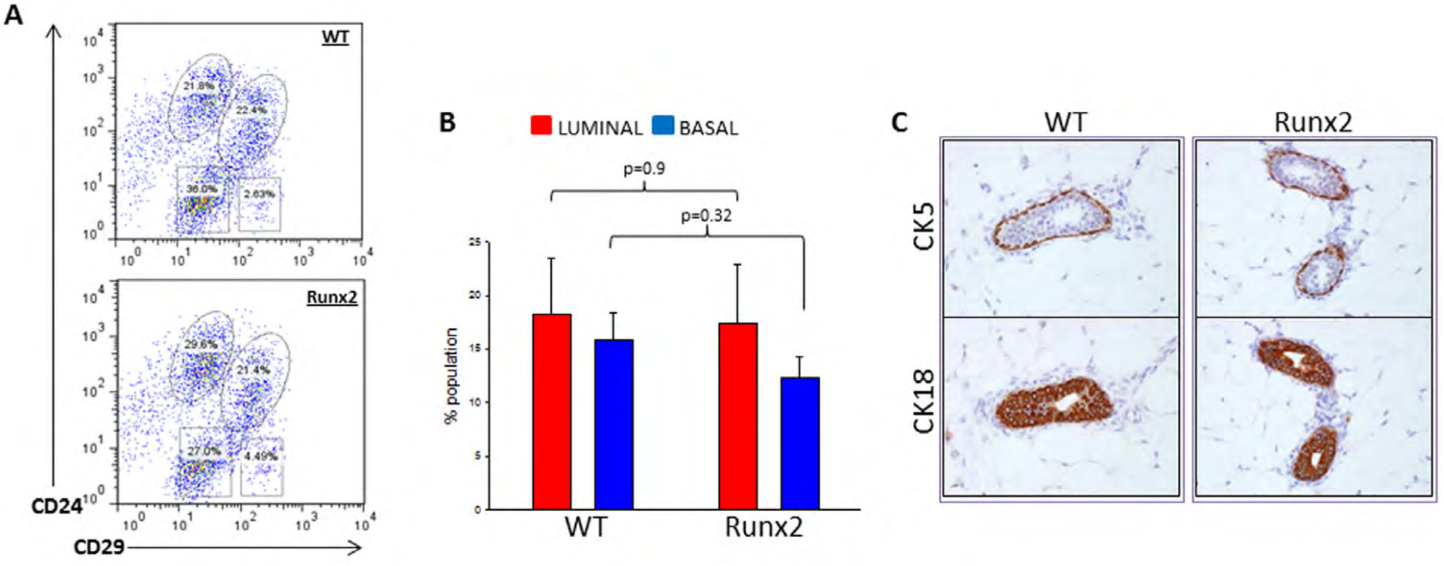

Supplementary Figure S3. Ectopic *Runx2* expression does not alter the ratio of luminal to basal/myoepithelial cells. (A) Representative flow cytometry profiles of whole mammary gland gated on *lin*$^-$ cells (DAPI$^-$/CD31$^-$/CD45$^-$) and assessed for CD29/CD24 marker expression. (B) The percentage of luminal and basal/myoepithelial cells was not significantly different between wild type (WT, n=11) and transgenic glands (*Runx2*, n=9). Data are means ±SD. (C) Representative immunohistochemistry for CK5 (basal) and CK18 (luminal) marker expression which is similar in both WT and Runx2 glands.

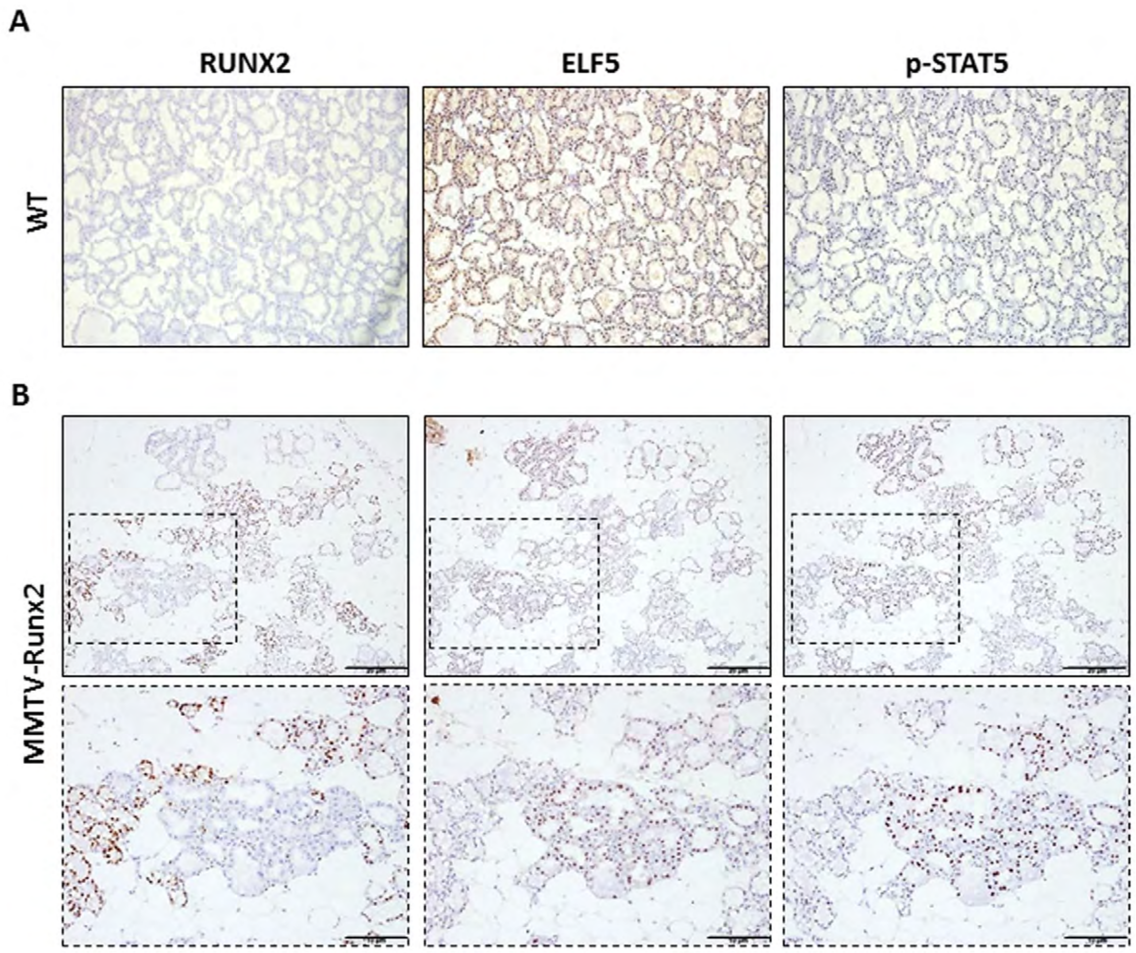

Supplementary Figure S4. ELF5/p-STAT5 expression is inhibited in *Runx2*-expressing alveolar tissue of multiparous transgenic glands. Serial sections of WT (A) and transgenic (B) multiparous lactating glands stained for RUNX2, ELF5 and p-STAT5 expression. Note reciprocal expression of RUNX2 and ELF5/p-STAT5 in areas of RUNX2-transgenic expression. Representative glands shown (of n=3 vs n=3), Boxed areas are shown at higher magnification below.

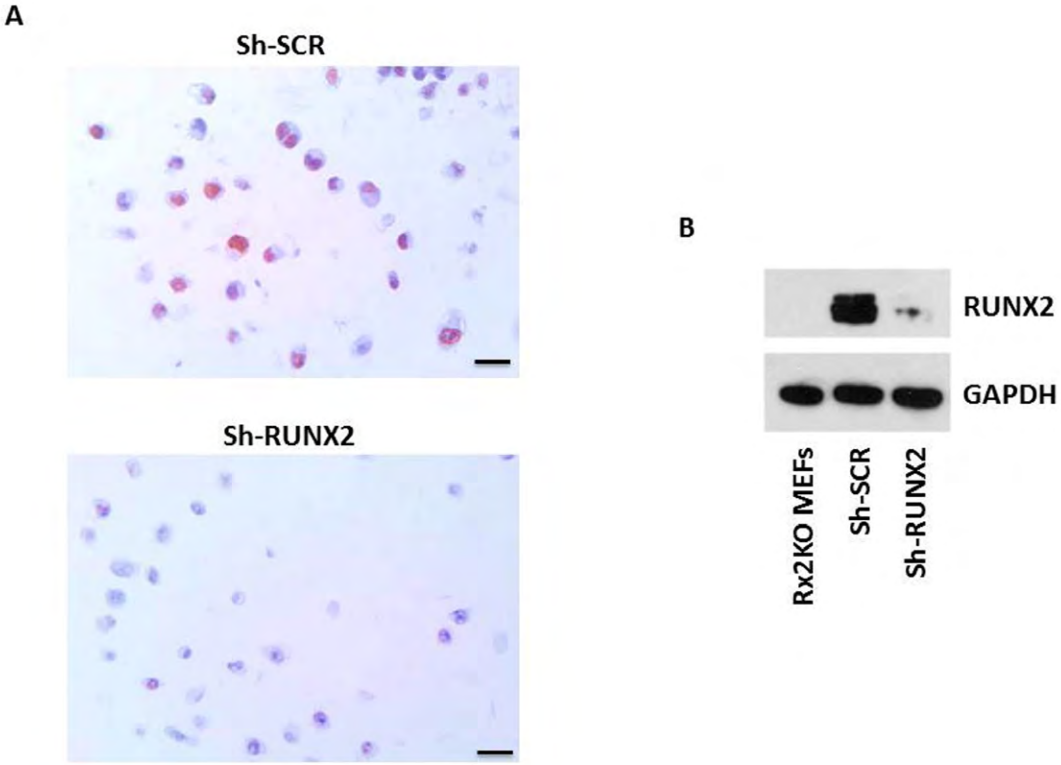

Supplementary Figure S5. Validation of the RUNX2 antibody. (A) RUNX2 antibody specificity was confirmed by immunohistochemistry on MDA-MB-231 cells transfected with a scrambled shRNA (sh-SCR) or an shRNA targeting RUNX2 (sh-RUNX2). Scale bars represent 10µm. (B) RUNX2 antibody specificity was further confirmed by western blot using MDA-MB-231 cells transfected with a scrambled shRNA (sh-SCR) or an shRNA targeting RUNX2 (sh-RUNX2). RUNX2 knock-out mouse embryonic fibroblasts (Rx2KO MEFs) were used as negative control. GAPDH was used as a loading control.
